# The sweet side of H5N1 influenza virus infection

**DOI:** 10.1371/journal.ppat.1012847

**Published:** 2025-01-23

**Authors:** Marina R. Good, Devika Suja, Jenna J. Guthmiller

**Affiliations:** Department of Immunology and Microbiology, University of Colorado Anschutz Medical Campus, Aurora, Colorado, United States of America; University of Wisconsin-Madison, UNITED STATES OF AMERICA

H5Nx viruses remain a threat to human health. Over the past few years, the H5Nx clade 2.3.4.4b has rapidly spread to 6 continents, leading to massive avian and mammalian host deaths. In late March 2024, H5N1 was first identified in lactating dairy cows in the United States and has spread to 16 states, affected hundreds of herds, and caused over 50 known human infections. In this review, we discuss the origins of 2.3.4.4b H5N1 viruses and how they are evolving to better infect mammals, with an emphasis on receptor-binding characteristics. Understanding changes in receptor binding and mutations in the viral genome that allow for sustained spread in mammals can inform public health measures and prevent future influenza virus epidemics and pandemics.

## What are avian influenza viruses and why are they concerning?

Avian influenza viruses circulate in birds, particularly waterfowl and shorebirds. Bird populations can carry high and low pathogenic avian influenza (HPAI and LPAI), which is based on the ability of the virus to cause severe or mild disease in birds, respectively. H5Nx viruses can be both HPAI or LPAI, with HPAI H5Nx (x referring to any NA subtype) viruses commonly having a polybasic cleavage site in the hemagglutinin (HA) protein. HPAI H5Nx clades threaten wild and domestic bird populations, as well as mammals and humans as a zoonotic virus [1]. Moreover, nearly 900 HPAI H5Nx infections in humans have been detected, with nearly half of those diagnosed succumbing to infection (approximately 50% case fatality rate) [2]. Therefore, HPAI pose a risk to ecosystems, agriculture, and human health globally. Since March 2024, H5N1 viruses within the 2.3.4.4b clade have caused an unprecedented outbreak in dairy cows in the United States of America. The spread and persistence of H5N1 viruses in mammals raises concerns of H5N1 adapting to mammalian hosts, which could lead to a pandemic in humans [1].

## What are 2.3.4.4b H5N1 viruses and where do they come from?

H5N1 viruses have been evolving over the last several decades and have diverged into different clades. The most recent dominantly circulating HPAI H5N1 clade is 2.3.4.4b, which is phylogenetically distinct from other H5N1 clades and is descended from 2.3.4.4 viruses [3]. The 2.3.4.4b clade also houses the B3.13 genotype viruses that are causing the current outbreak in dairy cows [4]. HPAI H5N1 clade 2.3.4.4b viruses were first detected in North America in late 2021 after introduction into the North American flyway [4]. They have caused significant mortality in wild bird populations and spillovers in domestic poultry, leading to the culling of millions of birds [3,4]. Phylogenetic analyses and epidemiological data have revealed that prior to circulating in the dairy cow population, the HPAI H5N1 genotype B3.13 viruses experienced a reassortment in wild birds, followed by likely a single transmission event between a bird and a dairy cow [4]. This reassortment resulted in the acquisition of new PB2 and NP genomic segments, likely from an LPAI late in 2023 [5]. The HPAI 2.3.4.4b viruses have also been able to successfully infect wild mammals and humans, exemplifying the ability of these HPAI H5N1 viruses to cross species barriers.

## Are H5N1 viruses changing their receptor specificity to better infect humans?

The entry receptor for most influenza A viruses is sialic acid (SA), a common terminal sugar of complex glycans found on the surface of host cells [6]. SAs can be composed of either N-acetylneuraminic acid or N-glycolylneuraminic acid (Neu5Ac and Neu5Gc, respectively). Neu5Ac is the SA receptor for H5Nx viruses, as most bird species do not express Neu5Gc [7]. Moving forward, the term SA will refer to Neu5Ac SAs. SAs link to the core glycan via either α2,3 or α2,6 linkages, and α2,3 linked SAs are preferred by avian influenza viruses (i.e., H5N1), while α2,6 linked glycans are preferentially bound by human seasonal viruses (i.e., H1N1, H3N2). HA binds to SAs via its receptor-binding site (RBS), which is highly adapted to either α2,3 or α2,6 SAs, with several important amino acid contacts that determine SA binding specificity. As the virus evolves, changes to these and other amino acids can be crucial in increasing SA binding breadth or switching SA receptor preference (H3 numbering).

**Fig 1:**
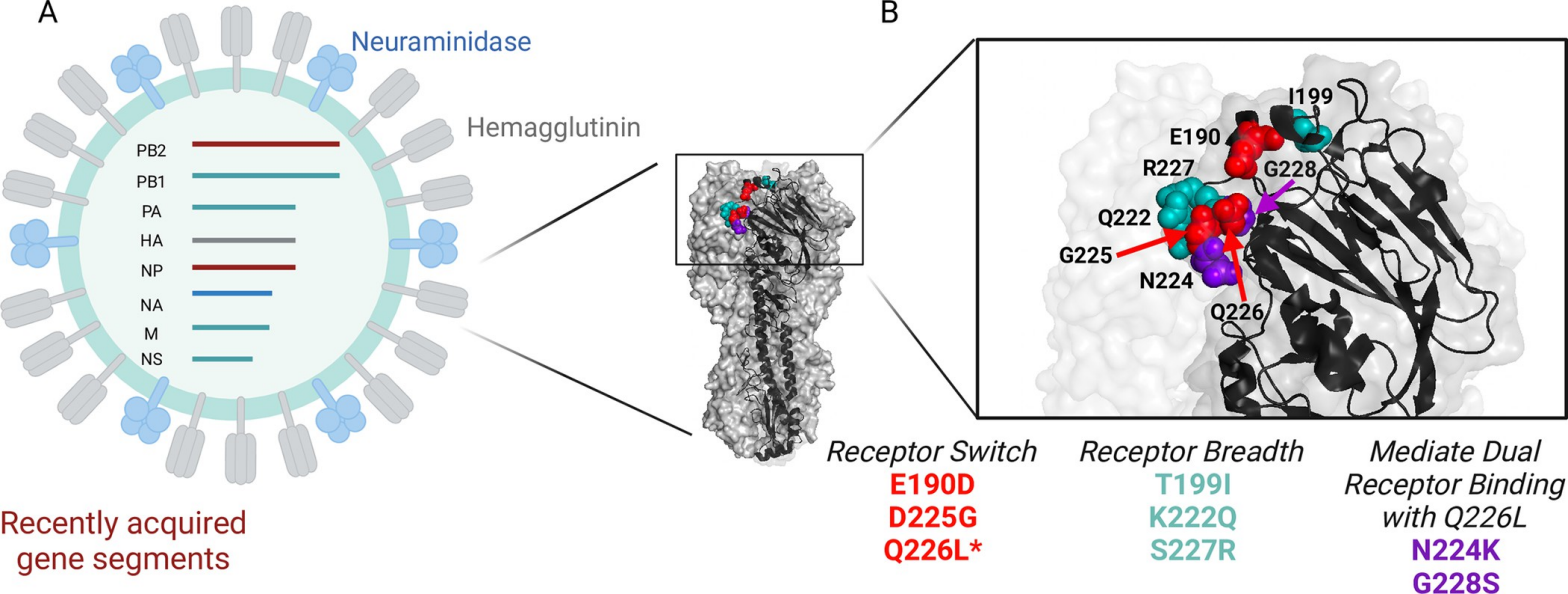
(A) Cartoon depiction of an influenza virion, the genomic segments, and surface glycoproteins. The segments are color coded to highlight recently acquired gene segments in the H5N1 reassortment that has given rise to the current dairy cow-associated epidemic. (B) Structure of the H5 head domain of the HA protein from A/Texas/37/2024. One monomer, shown as ribbon, illustrating residues that result in a sialic acid receptor-binding switch (red), those that result in changes in receptor-binding breadth (aqua), and those that promote α2,6 binding and mediate dual receptor binding (purple). PDB 9DWE. Both panels were created in part with BioRender.

- E190D and D225G are well-documented mutations that result in a switch from α2,3 to α2,6 SA binding (Fig 1) [3].
- Q226L can result in receptor switching to α2,6 SA in dairy cow-associated H5N1 viruses [8]. Q226L combined with N224K and/or G228S can improve α2,6 SA binding, while all 3 mutations together can mediate dual receptor binding to α2,3 and α2,6 SAs (Fig 1).
- 2.3.4.4 viruses acquired mutations K222Q and S227R in the mid-2010s, which increased binding to 3′ sialyl Lewis X glycans, which possess a bulky fucose group. 2.3.4.4b H5N1 viruses have retained these mutations and have retained a preference for 3′ sialyl Lewis X (Fig 1) [9].
- T199I has been recently described to broaden α2,3 SA binding in dairy cow-associated H5N1 viruses to both 3′ sialyl Lewis X and α2,3 sialylated N-acetyllactosamine (Fig 1) [3].

A recent study showed that an H5N1 virus isolate from the dairy cow outbreak has dual binding to α2,3 and α2,6 linked SAs [10]. However, multiple studies using recombinant HA or viruses from nearly identical isolates report highly specific binding of HA to α2,3 SAs, with no evidence of binding to α2,6 SAs [3,11]. These studies support the monitoring of SA-binding properties for pandemic risk assessment.

Other mutations can change viral transmission, immune evasion, and adaptation to mammalian hosts independent of receptor binding.

- Mutations within HA that increase stability are associated with increased airborne transmissibility; these mutations are concentrated in stalk domain α-helices and at the base of the head domain Y17H, A19T, H24Q, E31K, H110Y, and T318I (mature H3 numbering) [12].
- Mutations in nucleoprotein decrease the virus’ sensitivity to host restriction factor MxA, including I41T, R100V, R102A, L238P, F313Y, and Q399R [13].
- PB2, one of the proteins that forms the viral polymerase, has acquired mutations that evade innate immunity (I283M and K526R) and support infection of mammalian host cells (E627K, M631L, D701N, T271A, and Q591K) [4,14]. Notably, E627K, M631L, and D701N were increased in variants identified in the dairy cow-associated H5N1 2.3.4.4b outbreak [4].
- Neuraminidase (NA) is the other viral surface glycoprotein and cleaves sialic acid to allow virion release. Mutations (E119A and R156K) decrease NA enzymatic activity and are important for avian-to-human H5N1 adaptation [15].

## What hosts and tissues are SA receptors distributed across?

To better predict and prevent future zoonotic transmission events, a key area that needs to be investigated is how influenza viruses establish themselves in animal reservoirs. Receptor binding specificity and SA distribution are critical for host range, pathogenesis, and interspecies transmission of influenza viruses (Table 1) [16]. For example, α2,6-linked SAs are mainly concentrated in the upper respiratory tract of humans, particularly in the nasal epithelia and, while α2,3-linked SAs are more prevalent in the lower respiratory tract, such as the bronchi and alveoli [16]. The abundance of α2,6-linked SAs in the upper respiratory tract enables human influenza viruses to transmit more efficiently between individuals. Notably, α2,3-linked SA receptors are abundantly expressed in the upper airways and gastrointestinal tracts of birds [17]. Moreover, α2,3-linked receptors are highly present in the human cornea and conjunctiva [16]. This distribution of glycans likely explains the severe conjunctivitis reported as the primary clinical symptom in patients infected with HPAI H5N1 from the dairy cow epidemic in the United States [18]. The co-expression of both human and avian receptors in the pig respiratory tract raises concerns about zoonotic potential, as spillover of H5N1 into pigs could act as mixing vessels for novel influenza A virus reassortants [19].

**Table 1:** Distribution of distinct SA linkages in different hosts and tissues.

Species	SA distribution		
	Upper respiratory tract	Lower respiratory tract	Other relevant tissue
**Humans [16]**	α2,6	α2,3 > α2,6	Conjunctival epithelial cells - α2,3 and α2,6 Corneal epithelium - α2,3
**Pigs [19]**	α2,3 and α2,6	α2,3 ≈ α2,6	
**Ferrets [6]**	α2,3 < α2,6	α2,3 < α2,6	
**BALB/C Mice [6]**	α2,3 ≈ α2,6	α2,3 > α2,6	
**Dairy Cattle [20]**	α2,3 > α2,6	α2,3 > α2,6	Mammary gland - α2,3 >> α2,6
**Guinea Pigs [21]**	α2,3 ≈ α2,6	α2,3 > α2,6	
**Horses [6]**	α2,3 > α2,6	α2,3 > α2,6	
**Dogs [22]**	α2,3 > α2,6	α2,3 > α2,6	
**Chicken [23]**	α2,3 < α2,6		Intestinal epithelial cells - α2,3 > α2,6
**Ducks [17]**	α2,3 > α2,6		Intestinal epithelial cells - α2,3 > α2,6

The distribution of SAs in domestic, peridomestic, and wild animals and humans creates opportunities for viruses to easily cross species barriers and perhaps adapt to human hosts. Most human infections with avian influenza viruses are typically a result of direct and prolonged contact with infected animals. Additionally, cross-species infections are typically dead-end events due to a lack of viral adaptation necessary for efficient transmission. However, there are rare infections that lead to widespread outbreaks due to changes in infection kinetics, immune evasion, and receptor preference, emphasizing the ability of influenza to adapt quickly to new host and tissue environments.

## Conclusions and outstanding questions

SA binding preference plays a major role in determining the host and tissue tropism of influenza viruses. The α2,3 SA binding preference of H5N1 viruses remains a major barrier for sustained transmission in humans. While canonical mutations associated with α2,3 to α2,6 SA binding have not been observed in 2.3.4.4b H5N1 viruses, numerous questions remain about the capacity of these viruses to evolve to infect new hosts and potentially cause a human pandemic.

1. Are there uncharacterized mutations that could confer both α2,3 and α2,6 binding?
2. Are there other mutations that confer a receptor switch from α2,3 and α2,6 SA preference?

There is a need for continued surveillance to inform public health preparedness. There are many questions that remain unanswered about zoonotic potential, including viral evolutionary patterns, host factors that restrict or permit H5N1 infection, and how H5N1 transmits between and across different animal species.

1. How does the distribution of glycans on host tissues influence viral adaptation and evolution?
2. Are there sequence determinants involved in receptor binding changes that can be detected in early emerging viruses?
3. Are there ways that receptor binding and breath can be surveyed to assess the risk of potential pandemic influenza viruses?

Answers to these questions would help predict zoonotic potential, assess pandemic risk, guide vaccine development, improve surveillance, and enhance public health preparedness.
